# Ecological study of ambient air pollution exposure and mortality of cardiovascular diseases in elderly

**DOI:** 10.1038/s41598-022-24653-0

**Published:** 2022-12-09

**Authors:** Samaneh Dehghani, Mohebat Vali, Arian Jafarian, Vahide Oskoei, Zahra Maleki, Mohammad Hoseini

**Affiliations:** 1 Department of Environmental Health Engineering, School of Public Health, Tehran University of Medical Sciences, Tehran, Iran; 2 Department of Epidemiology, School of Health, Shiraz University of Medical Sciences, Shiraz, Iran; 3 Department of Environmental Health Engineering, School of Health, Shiraz University of Medical Sciences, Shiraz, Iran; 4 Department of Environmental Health Engineering, School of Public Health, Research Center for Health Sciences, Institute of Health, Shiraz University of Medical Sciences, Razi Blvd, Kuye-Zahra Ave, Shiraz, 1417653861 Iran

**Keywords:** Ecological epidemiology, Environmental sciences

## Abstract

As an independent risk factor, ambient air pollution can assume a considerable part in mortality and worsening of cardiovascular disease. We sought to investigate the association between long-term exposure to ambient air pollution and cardiovascular disease mortality and their risk factors in Iranian's elderly population. This inquiry was conducted ecologically utilizing recorded data on cardiovascular disease mortality from 1990 to 2019 for males and females aged 50 years or more from the Global Burden of Disease dataset. Data was interned into Joinpoint software 4.9.0.0 to present Annual Percent Change (APC), Average Annual Percent Change (AAPC), and its confidence intervals. The relationship between recorded data on ambient air pollution and cardiovascular disease' mortality, the prevalence of high systolic blood pressure, high LDL cholesterol levels, high body mass index, and diabetes mellitus type2 was investigated using the Spearman correlation test in R 3.5.0 software. Our finding demonstrated that cardiovascular diseases in elderly males and females in Iran had a general decreasing trend (AAPC = −0.77% and −0.65%, respectively). The results showed a positive correlation between exposure to ambient ozone pollution (p ≤ 0.001, r = 0.94) ambient particulate and air pollution (p < 0.001, r = 0.99) and mortality of cardiovascular disease. Also, ambient air pollution was positively correlated with high systolic blood pressure (p < 0.001, r = 0.98), high LDL cholesterol levels (p < 0.001, r = 0.97), high body mass index (p < 0.001, r = 0.91), diabetes mellitus type2 (p < 0.001, r = 0.77). Evidence from this study indicated that ambient air pollution, directly and indirectly, affects cardiovascular disease mortality in two ways by increasing the prevalence of some traditional cardiovascular disease risk factors. Evidence-based clinical and public health methodologies are necessary to decrease the burden of death and disability associated with cardiovascular disease.

## Introduction

Exposure to air pollution increments morbidity and mortality of cardiovascular disease (CVD)^1^, especially exposure to emissions from traffic and industrial sources^2^. Even lower concentrations of exposure to particulate matter (PM) caused by combustion, as a significant component of urban pollution, are involved in the pathogenesis of CVD^3^. Some researchers have shown a relationship between intense or persistent exposure to PM and the rate of cardiopulmonary occasions^4^. Increased ambient air pollution (AAP) can be considered a risk factor for heart failure^5^, myocardial infarction, cardiovascular stroke^6^, and death^7,8^. These effects can be due to intense daily variation in air pollutant levels just as lifelong exposure to them^1^. Long-term exposure to PM air pollution per 10 μg/m^3^ is estimated to increase the overall mortality rate by 2–4%, with the highest mortality rate from CVD^2^.

Although the etiology underlying this long-term relationship is unknown^9^, Some pathways are considered as possible biological mechanisms for increased risk of CVD incidence due to air pollution exposure. Disorders of the autonomic nervous system of the heart, pulmonary and systemic oxidative stress and inflammatory responses that impair endothelial function, atherosclerosis, and thrombosis have been reported as direct effects of AAP^10^. Long-term air pollutants exposure at low levels participates in hypertension^11^, obesity^12,13^, and diabetes^14^, which are involved in the CVD epidemiology^15^. Hence, AAP indirectly affects CVD risk through changes in blood pressure, triglyceride, blood sugar, and vascular functions^16^.

Iran's aging population will be forecasted to be 10.5% in 2025 and 21.7% in 2050, following the oldest population in the region by 2050^17^. Despite the medical advances in treatment and control of CVD, which have prompted a significant decrease in CVD rate, prevention is still considered a priority, especially to reduce clinical expenses^18,19^. Due to the vulnerability of the cardiovascular system to environmental factors reported in previous studies in other earas^20,21^, this ecological research was performed aimed to analyze 1- the relationship between AAP exposure and CVD mortality as the first cause of death in Iran, 2- the relationship between exposure to AAP and the prevalence of traditional risk factors for CVD, including diabetes, hypertension, obesity, and high LDL cholesterol in Iranian over 50 years of age in 1990–2019.

## Materials and methods

### Data collection

Cardiovascular diseases (CVDs), principally ischemic heart disease (IHD) and stroke, are the leading cause of global mortality and a major contributor to disability. Data on CVD mortality was collected separately for each year from 1990 to 2019 in Iran based on sex and age groups of 50–69 years and over 70 years^22,23^. In addition, the prevalence of traditional CVD risk factors collected by year and in the age and sex groups of 50–69 years and over 70 years, tobacco use, smoking, diabetes mellitus type 2 (DM2), high LDL cholesterol, high body mass index (BMI), and high systolic blood pressure (SBP). The definition of the elderly group with a reference for considering 50 years old as a cut point of age for the elderly group was considered.

The blood low-density lipoprotein (LDL) concentration is estimated in mmol/L, and the theoretical minimum risk exposure level value (TMREL) is used with a uniform distribution between 0.7 and 1.3 mmol/L. The values of brachial SBP are estimated in millimeters of mercury, and TMREL SBP 110 to 115 mm of mercury is used. DM is characterized collectively as a metabolic disease whose common component is a raised blood glucose level (hyperglycemia). Also, adults' high BMI (ages 20 and up) is characterized as a BMI of more than 20–25 kg body weight in the second power of height (meters)^22^. Air pollution exposure in this study is defined as ambient particulate air pollution (PM), ambient ozone pollution, and air pollution (the sum of PM and ozone pollution) based on the Global Burden of Disease (GBD) categorization in micrograms per cubic meter (µg/m^3^). Satellite data has been used to measure air pollution^22^.

GBD dataset uses many different sources to populate the information in the GHDx. Most information about datasets and series comes from the data providers; also was used many of the resources are noted on the Data Sites We Love page, particularly are indebted to organizations such as IPUMS, ICPSR, and the World Bank. For older data, WorldCat is an invaluable resource. All of the data is collected from the following official websites: http://www.healthdata.org/.

Based on the GBD 2019 study, this site covers injuries and risk factors from 1990 to 2019, covering 204 countries and territories. In total, 369 causes of illness and injury were systematically analyzed. The methodology of the GBD studies and the principal changes incorporated into the GBD 2019 method has been explained in detail elsewhere^24^.

### Statistical analysis

Excel software was used for preliminary analysis, including graphs and maps. Then the information was prepared to enter the joinpoint software. Descriptive analysis for CVD mortality was performed using the regression model in the Joinpoint software (version 4.9.0.0). The joinpoint software is one of the statistical software available by the American Cancer Society to perform joinpoint regression. The latest version of the software was used to perform the regression analysis of attachment points. The regression of attached points actually forms segments by creating statistically significant points compared to the previous point, and for each segment or piece, an APC (annual percentage change) or annual percentage change will be created. In addition, the software provides average annual percentage change or AAPC (Average Annual percentage change). With these two values, the trend of changes from 1990 to 2019 was investigated. We reported the APC, AAPC, and related confidence intervals in the Iranian male and female populations over 50 years old separately. The Descriptive results of the PM data section, the mortality data related to ambient particulate matter pollution exposure, particulate matter pollution exposure, and air pollution exposure are reported separately in the method section of the jointpoint regression model as well as deathes due to the CVD. Spearman's correlation was utilized to investigate the relationships between ambient air pollution data and specific CVD mortality rates and the prevalence of high SBP, high BMI, DM2, high LDL cholesterol, and other risk factors in the R software (version 3.5.0).

**Figure 1:**
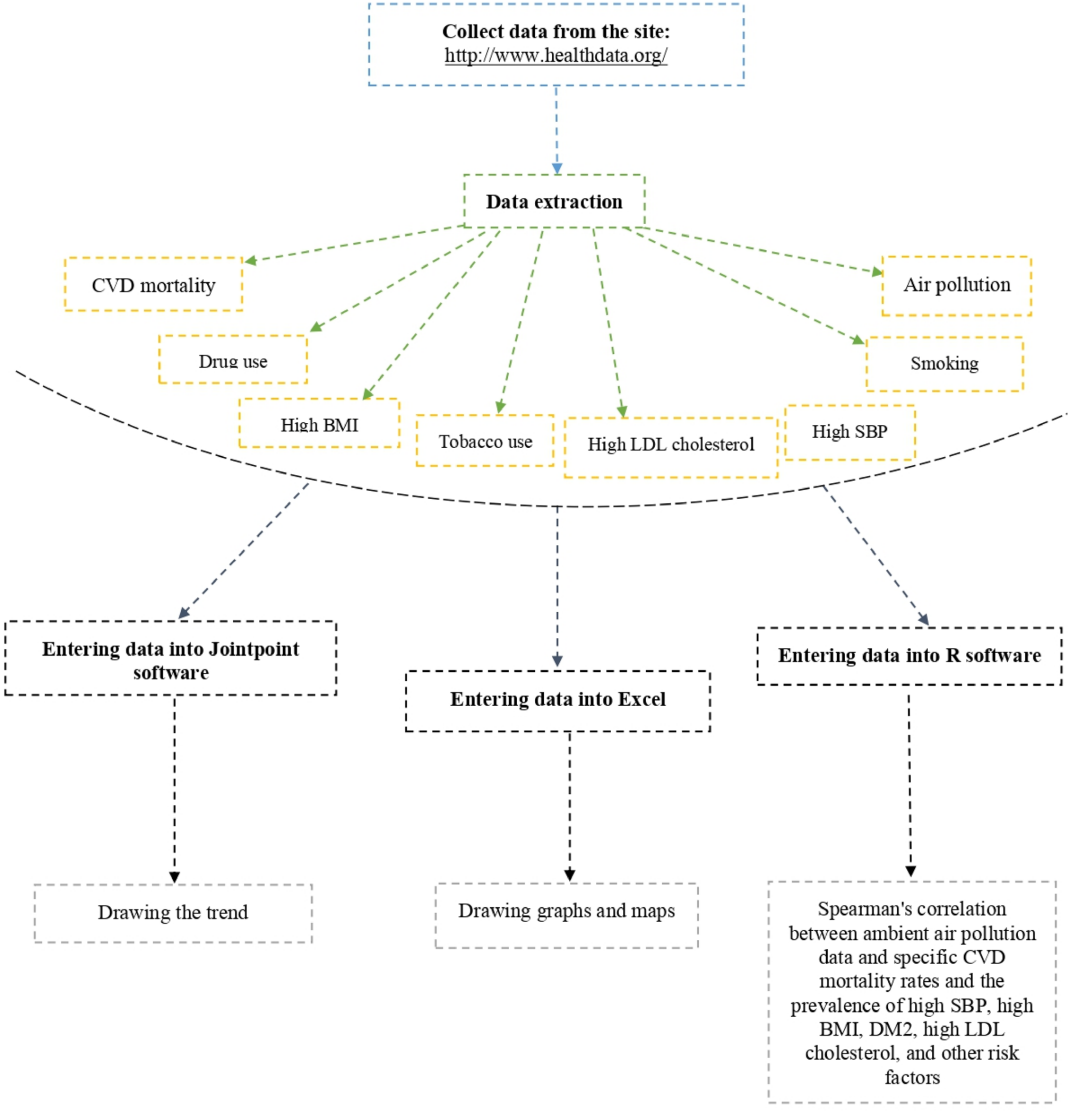
Detail of the plan of the study.

Correlations of air pollution-related mortality or prevalences for each corresponding year are analyzed. All statistical analyses were two-sided, and a p-value less than 0.05 (p < 0.05) was considered significant. Correlation intensity was interpreted based on correlation values; r = 0.8–1 very strong correlation, r = 0.6–0.08 strong correlation, r = 0.4–0.6 moderate correlation, r = 0.2–0.4 low or weak correlation relationship^25^. The detail of the plan of the study was shown in the Fig. 1.

## Results and discussion

One of the most controversial issues related to climate is urban development and its pollution. Researches on air pollutants and climatic elements suggest a link between the two factors and the mortality and incidence of disease. The extent to which the climate effect the severity of many diseases, including lung, heart, infectious and contagious diseases, is undeniable^26^. Cardiovascular failure is a major general health problem that influences over 23 million people worldwide, with a 1-year mortality rate of 30% and a rate of annual hospitalization of 2%. Recently, AAP has been considered a short-term trigger in developing heart failure^27–30^. The present study uses recorded data on mortality of the CVD and risk factors for this disease (DM2, high LDL cholesterol, high BMI, and high SBP) in age groups above 50 years and its relationship with recorded data from air pollution from 1990 to 2019.

### Descriptive results of CVD mortality data

**Figure 2:**
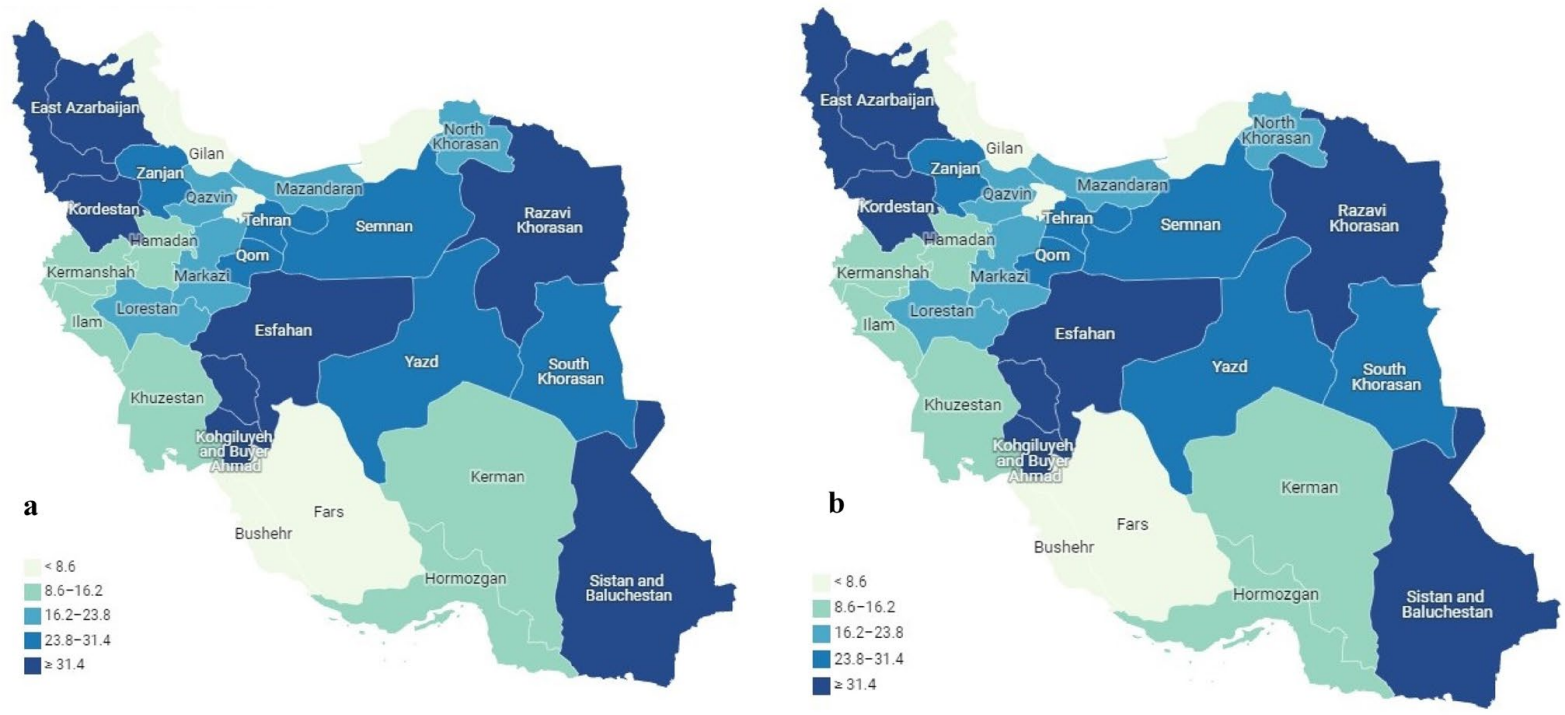
Distribution of deaths due to CVD per 100,000 by provinces of Iran in 1990 (**a**) and 2019 (**b**).

Deaths distribution of ischemic heart disease in 1990 and 2019 by provinces of Iran per 100,000 was shown in Fig. 2.

**Figure 3:**
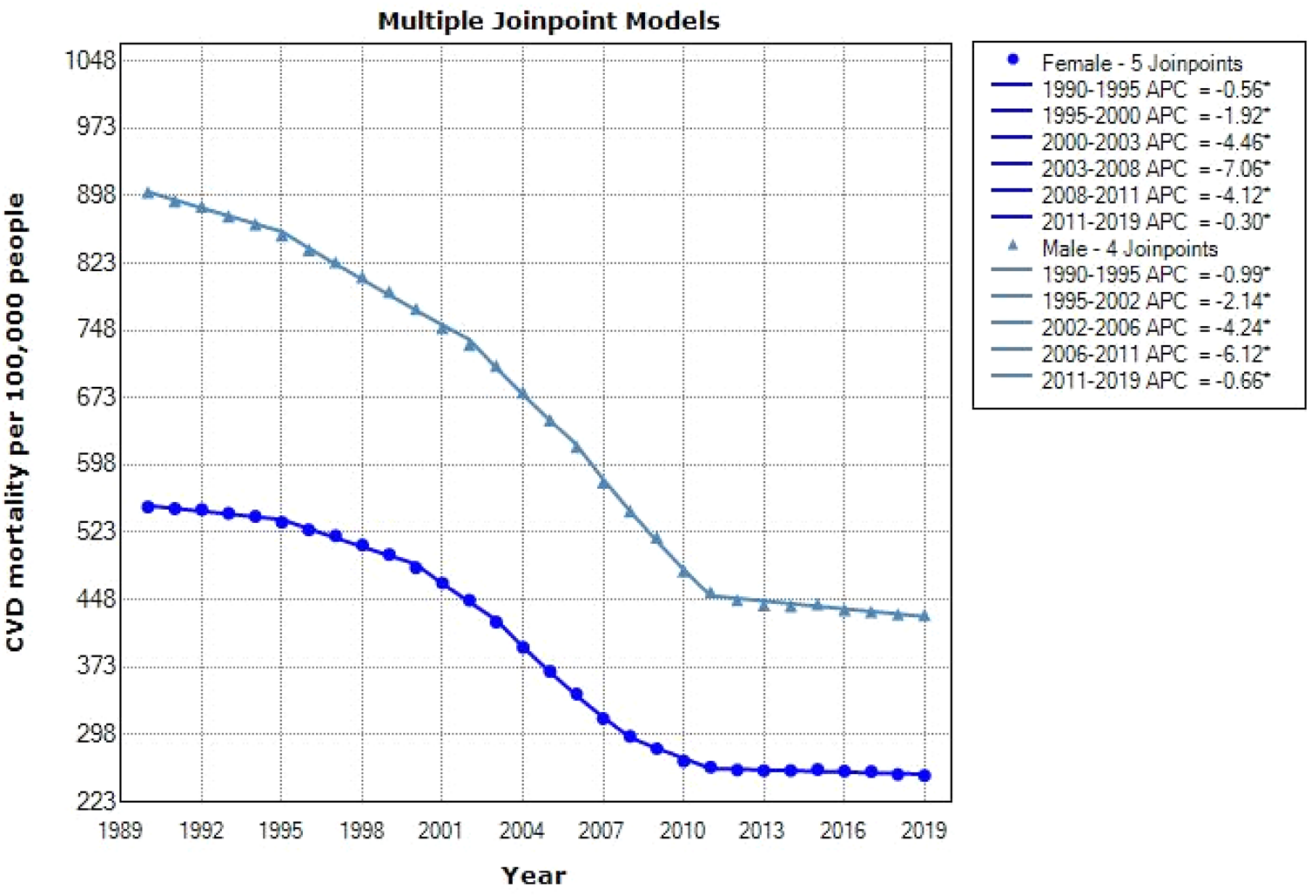
Jointpoint regression model for CVD mortality aged 50–69 by gender: 1990–2019.

**Figure 4:**
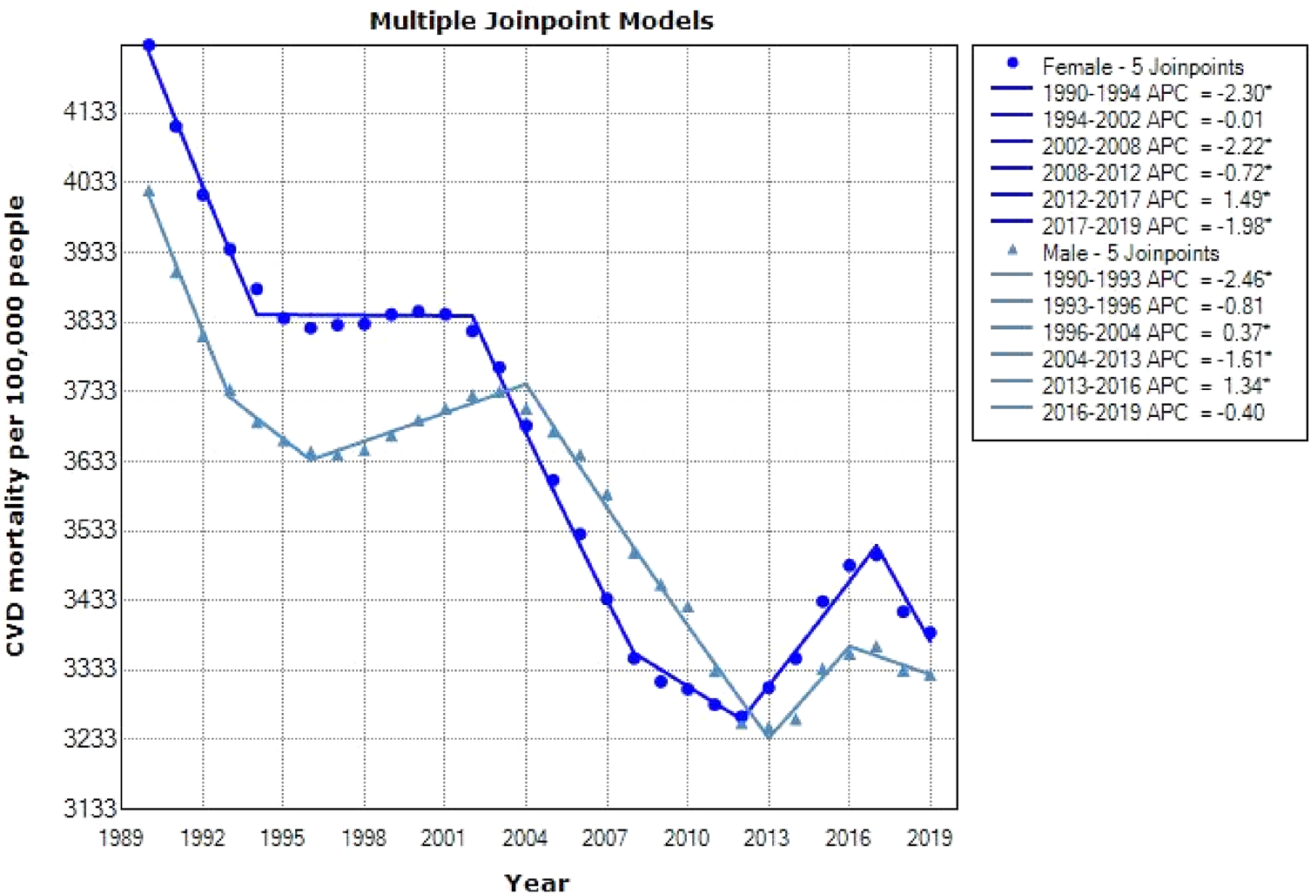
Joinpoint regression model for CVD mortality aged +70 by gender: 1990–2019.

Descriptive results of CVD mortality data from 1990 to 2019 in people 50–69 years old and over 70 years by gender were shown in Figs. 3 and 4, respectively. These results indicate that the mortality resulting from CVD in males 50–69 years and over 70 years had a downward trend [AAPC = −2.6% (CI: −2.8 to −2.4), AAPC = −2.5% (CI: −2.6 to −2.4), respectively] and also had a descending direction in females ranges 50–69 years and above 70 years old [AAPC = −0.7% (CI: −0.8 to −0.6), AAPC = −0.6% (CI: −0.8 to −0.4), respectively] (see in Table 1). The latest GBD study estimates that in 2019, there were 8.76 million disability-adjusted life years (DALYs, 146,000 deaths among females, and 131,000 deaths among males due to cardiovascular and circulatory diseases^31^.

**Table 1:** Descriptive statistics of CVD mortality are separated by gender and age.

Cohort		Joinpoints	Range	Lower endpoint	Upper endpoint	AAPC	Lower CI	Upper CI
50–69 years	Female	5	Full range	1990	2019	−2.65	−2.86	−2.44
	Male	4	Full range	1990	2019	−2.53	−2.65	−2.40
+70 years	Female	5	Full range	1990	2019	−0.77	−0.85	−0.68
	Male	5	Full range	1990	2019	−0.65	−0.84	−0.45

### Descriptive results of PM data

**Figure 5:**
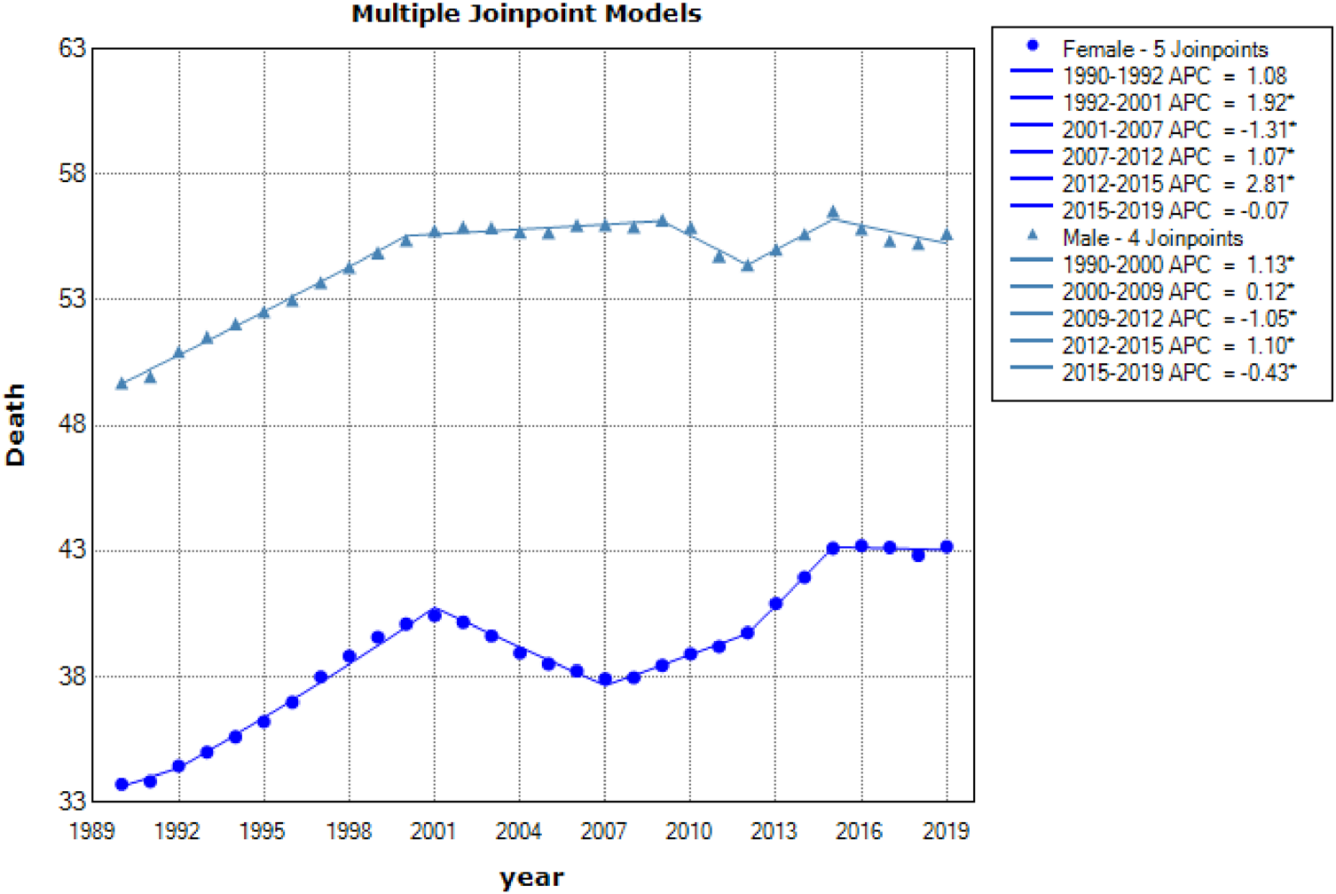
Joinpoint regression model for death due to ambient particulate matter exposure in aged +50 by gender: 1990–2019.

**Figure 6:**
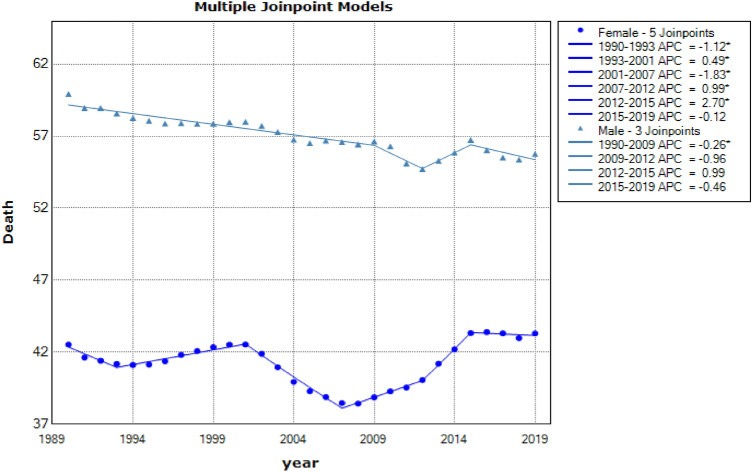
Joinpoint regression model for death due to particulate matter exposure in aged +50 by gender: 1990–2019.

**Figure 7:**
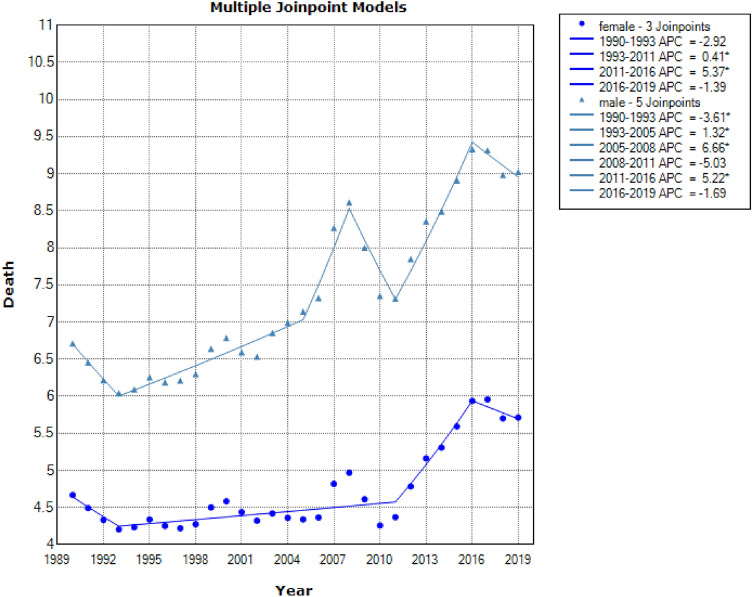
Joinpoint regression model for death due to ambient ozone exposure in aged +50 by gender: 1990–2019.

**Figure 8:**
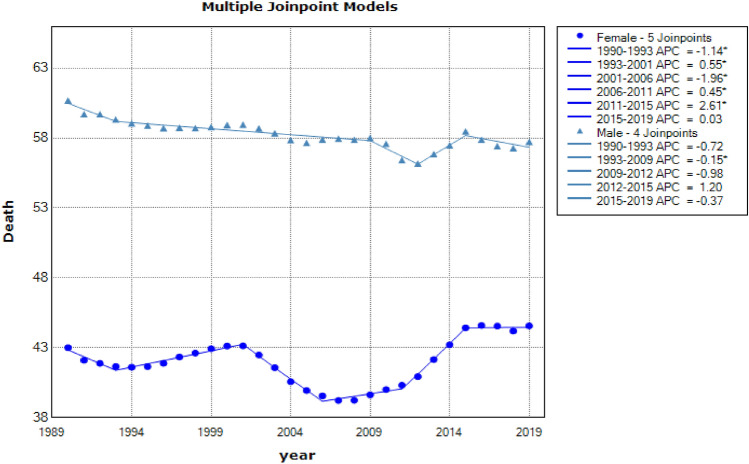
Joinpoint regression model for death due to air pollution exposure aged +50 by gender: 1990–2019.

Descriptive results of mortality data data on ambient particulate matter exposure, particulate matter pollution, ambient ozone exposure, and air pollution exposure from 1990 to 2019 in over 50 years males and females were shown in Figs. 5, 6, 7 and 8, respectively.

These results showed that mortality due to exposure to ambient particulate matter pollution in females over 50 years had an uptrend trend [AAPC = 0.85% (CI: 0.7 to 1.01), respectively] and also had an ascending direction in males over 50 years [AAPC = 0.37% (CI: 0.23 to 0.50), respectively] (see in Table 2).

**Table 2:** Descriptive statistics of mortality due to ambient particulate matter exposure separated by gender.

Cohort		Joinpoints	Range	Lower endpoint	Upper endpoint	AAPC	Lower CI	Upper CI	Test statistic ~	P-value ~
+ 50 years	Female	5	Full range	1990	2019	0.85	0.70	1.01	10.57	< 0.001
	Male	4	Full range	1990	2019	0.37	0.23	0.50	5.35	< 0.001

Mortality due to exposure to particulate matter in females over 50 years had an ascending direction [AAPC = 0.07% (CI: −0.1 to 0.23), respectively] and had a downward trend in males over 50 years [AAPC = −0.23% (CI: −0.46 to 0.0), respectively] (see in Table 3).

**Table 3:** Descriptive statistics of mortality due to particulate matter exposure separated by gender.

Cohort		Joinpoints	Range	Lower endpoint	Upper endpoint	AAPC	Lower CI	Upper CI	Test statistic ~	P-value ~
+ 50 years	Female	5	Full Range	1990	2019	0.07	−0.10	0.23	0.77	0.440
	Male	4	Full Range	1990	2019	−0.23	−0.46	0.00	−1.92	0.055

Mortality due to exposure to ambient ozone in females an males over 50 years had an ascending trend [AAPC = 0.71% (CI: −0.25 to 1.68)] 50 years [AAPC = 1% (CI: 0.12 to 1.90)], respectively (see in Table 4).

**Table 4:** Descriptive statistics of mortality due to ambient ozone exposure separated by gender.

Cohort		Joinpoints	Range	Lower endpoint	Upper endpoint	AAPC	Lower CI	Upper CI	Test statistic ~	P-value ~
+ 50 years	Female	3	Full range	1990	2019	0.71	−0.25	1.68	1.45	0.148
	Male	5	Full range	1990	2019	1.00	0.12	1.90	2.22	0.027

Finally, the mortality resulting from exposure to air pollution in males over 50 years had a downward trend [AAPC = −0.18% (CI: −0.4 to 0.04), respectively] and had a uptrend in females over 50 years [AAPC = 0.13% (CI: 0.0 to −0.26), respectively] (see in Table 5).

**Table 5:** Descriptive statistics of mortality resulting from air pollution exposure are separated by gender.

Cohort		Joinpoints	Range	Lower endpoint	Upper endpoint	AAPC	Lower CI	Upper CI	Test statistic ~	P-value ~
+ 50 years	Female	5	Full range	1990	2019	0.13	0.26	1.97	0.049	0.00
	Male	4	Full range	1990	2019	−0.18	0.04	−1.65	0.099	−0.40

Recently, air pollution has become a significant phenomenon, especially in developing countries, due to the increase of vehicles, congested traffic, inappropriate control of the release of pollutants from the resources, industrialization, and improper laws^32,33^. So, in this situation, air pollution has an increasing trend^34^. Particulate matter (PM), which was emitted due to biogenic and anthropogenic sources and produced by atmospheric reactions, is deliberated as the averse airborne pollutants^35^. Currently, West Asia is affected by dust storms in deserts, raising the number of dusty days and PM's daily average (PM_10_)^36,37^. PM_10_ can penetrate thoroughly into the airways and cause severe health effects on humans^38,39^.

Particulate air pollution and its harmful health impacts are not newborn dilemmas^40^. Recently, scientists have examined what characteristics of ambient aerosol caused the health impacts and whether chemical components of specific particulate have higher health effects than others^41^. There is a significant relation between dust events and daily hospitals because of respiratory illnesses in Asia^42–44^. Epidemiological inquiries have illustrated the detrimental impacts of PM air pollution on cardiorespiratory conditions^45,46^. Moreover, PM exposure has caused oxidative stress and systemic inflammation, as mentioned in some research^47,48^. Therefore there is an authoritative relationship between ambient exposure to air pollution and cardiopulmonary morbidity and mortality^48,49^.

### Ambient air pollution exposure and CVD mortality

**Figure 9:**
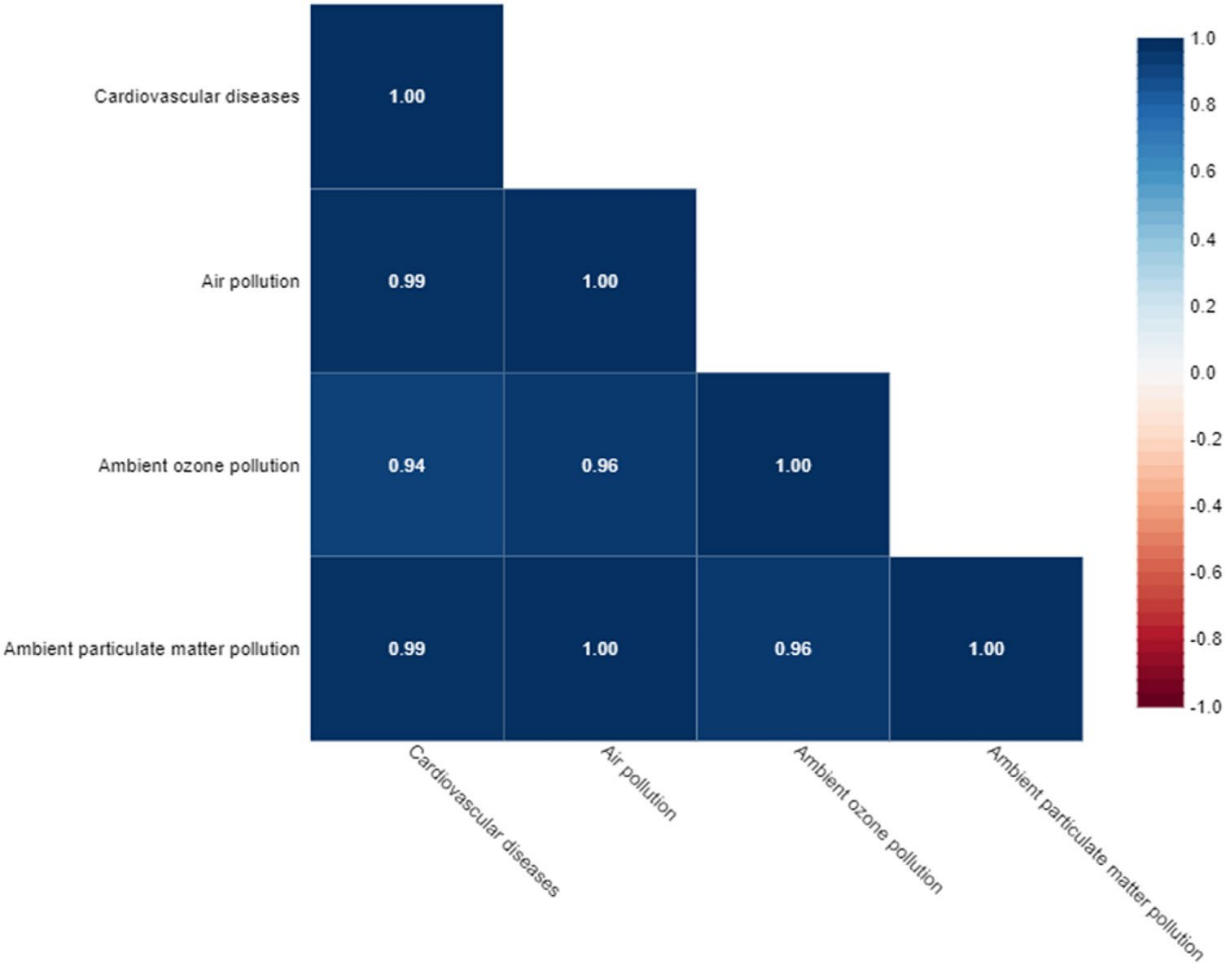
Spearman's correlation between CVD mortality and ambient air pollution (ambient PM and ozone pollution).

The relationship between CVD mortality and AAP levels is shown in Fig. 9. There was significant relationship and positive correlation between CVD mortality and ambient PM (p ≤ 0.001, r = 0.99), ambient ozone pollution (p ≤ 0.001, r = 0.94), and ambient air pollution (p ≤ 0.001, r = 0.99) in over 50 years adults in Iran.

Brook et al.'s research estimated that 69% of early deaths result in CVD, comprising ischemic heart disease and stroke, while lung disease accounts for only 28%^7^. Evaluations in the GBD Scheme showed that AAP greatly affects mortality more than any other important modifiable factor, including low physical activity and elevated cholesterol and sodium diets^50^. Numerous manifestations of CVD are related to air pollution, including arterial and venous circulation. Air pollution exposure additionally appears to assume a significant part in disease progression. Incredibly, there is ample evidence of PM's adverse effects on cardiovascular health compared to gaseous pollutants^51^. Significant effects of PM_2.5_ were obtained from long-term exposure that affects mortality from CVD^52^. The risk of CVD following exposure to PM2.5 was higher in men, the elderly, and those with hypertension, diabetes, heart disease, or background of stroke^53^. Findings of the Thurston et al. study reported that PM2.5 exposure for a long course increases total risk and CVD risk in this cohort of subjects^54^. Some studies mention the rapid effect of exposure to air pollutants on the cardiovascular system^53,55,56^. The apparent link between air pollution and cardiovascular failure's systematic review and meta-analysis demonstrated that the transient rise in gas and particulate components was positively associated with hospitalization risk or death from congestive heart failure. People with persistent heart failure, hypertension, and arrhythmias are at a higher risk^5^. Several links have recently been recorded between out-of-hospital cardiac arrest and air pollution, mainly PM and ozone^57^.

Air pollution with directly and/or indirectly biological pathways affects CVD. Direct impacts of air pollution give a conceivable clarification to the event of fast cardiovascular reactions. For example, gases with PM_2.5_ soluble substances directly pass through the pulmonary epithelium into the bloodstream^58^ and raise the risk of ventricular fibrillation, myocardial infarction, and cardiac arrest by having a direct but weak effect on ventricular arrhythmogenesis^59^. PM_2.5,_ due to its small size, allows reaching the airways and small alveoli. Studies have shown that short-term exposure to PM boosts the rate of proinflammatory mediators in the blood, coagulation, and inhibition of fibrinolytic capacity^60,61^. Diesel exhaust exposure also causes inflammation within the plaque, changes in vasomotor tone, and inflammatory mediators^62^.

Among several metabolisms associated with air pollution and CVD mortality, the significant paths are induction of oxidative stress, systemic inflammation, endothelial function, atherosclerotic function, and arrhythmogenesis^63^. A decrease in PM_2.5_ concentrations is associated with a decrease in the progression of Antima-carotid thickness, which indicates biological viability^64^. High levels of ambient PM2.5 increase plaque load and vascular dysfunction in atherosclerotic mouse models^65,66^. Therefore, interpreting the positive relationship between PM2.5 exposure and CVD burden may be significant and support the biological association between air pollution exposure and atherosclerosis. On the other hand, investigations on the relationship between air pollution and CVD have yielded clashing outcomes. Several studies have demonstrated significant relevance^26–28,67,68^, while others were unrelated^69^.

### Prevalence of traditional risk factors and CVD mortality

**Figure 10:**
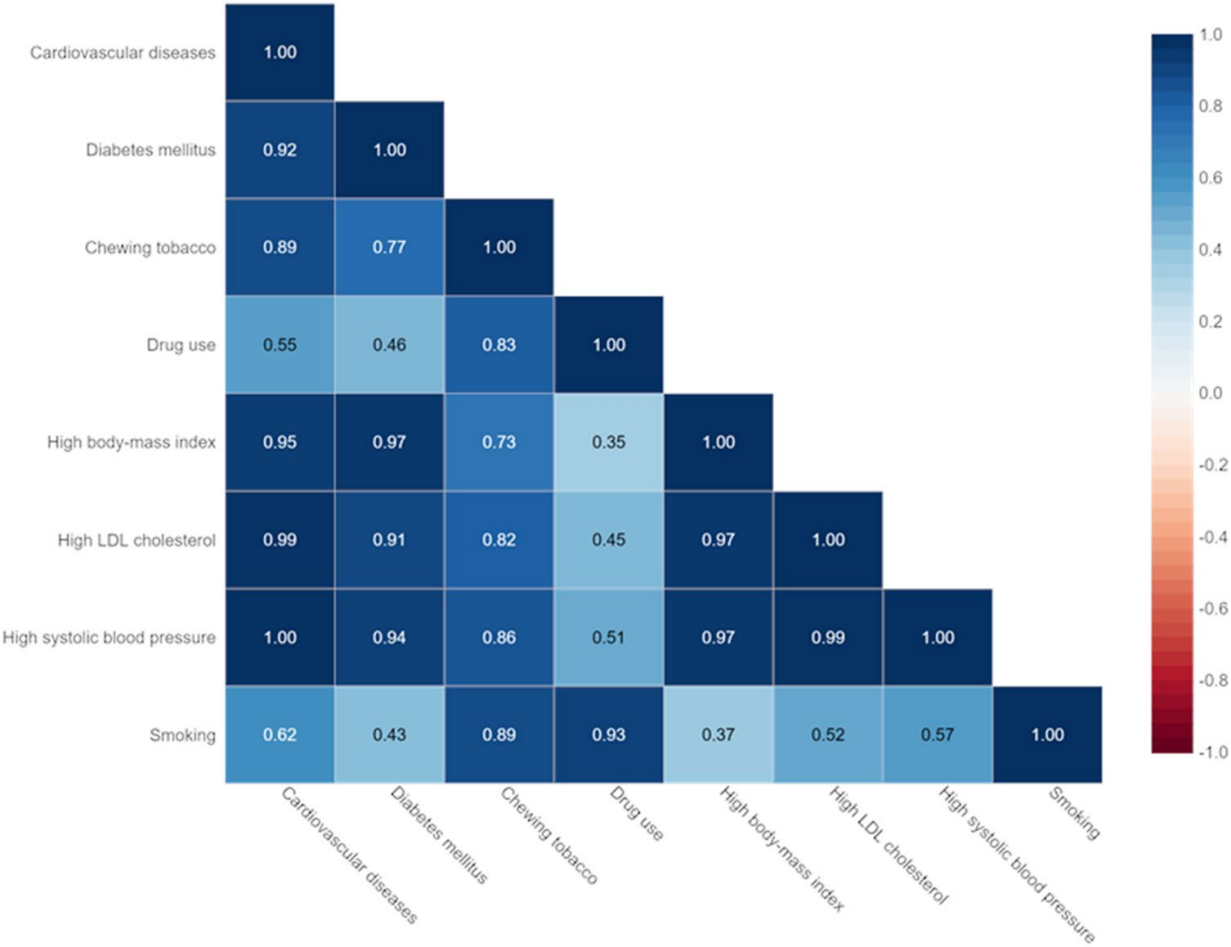
Relationship between CVD mortality and prevalence of CVD risk factors (DM2, tobacco and drug use, high BMI, high LDL cholesterol, high SBP, and smoking).

The relationship between CVD mortality in the Iranian population over 50 years and the prevalence of traditional CVD risk factors, including DM2, tobacco use, high BMI, high LDL cholesterol, high SBP, and smoking, are shown in Fig. 10. Results of Spearman correlation analysis, indicated significant relationship and positive correlation between high SBP (p ≤ 0.001, r = 1), high LDL cholesterol (p ≤ 0.001, r = 0.99), high BMI (p ≤ 0.001, r = 0.95), DM2 (p ≤ 0.001, r = 0.92), tobacco use (p ≤ 0.001, r = 0.89) and moderate positive correlation between smoking (p ≤ 0.001, r = 0.62) and tobacco use (p ≤ 0.001, r = 0.55) and the risk of death from CVD. According to the results of our study, DM2, high SBP, high levels of LDL cholesterol, and high BMI were important risk factors for CVD mortality.

CVD's risk assessment in an individual is usually conducted by CVD's traditional risk factors and subsequently anticipated using fully accessible algorithms, such as the Framingham Risk Score (FRS)^70^. Some of the most common metabolic consequences of fat, such as high blood pressure, diabetes mellitus, dyslipidemia, and obesity, were considered the principal risk factors for CVD (Bays et al., 2021). Also, In past texts, it has been well established that smoking and physical inactivity are among the risk factors for CVD^71–74^.

It remains to be seen, despite the widespread exposure to AAP and the relationship between AAP and traditional CVD risk factors, How important is this exposure to ambient air pollution on the prevalence, exacerbation, and mortality of CVD directly or indirectly?

### Ambient air pollution exposure and the prevalence of CVD risk factors

**Figure 11:**
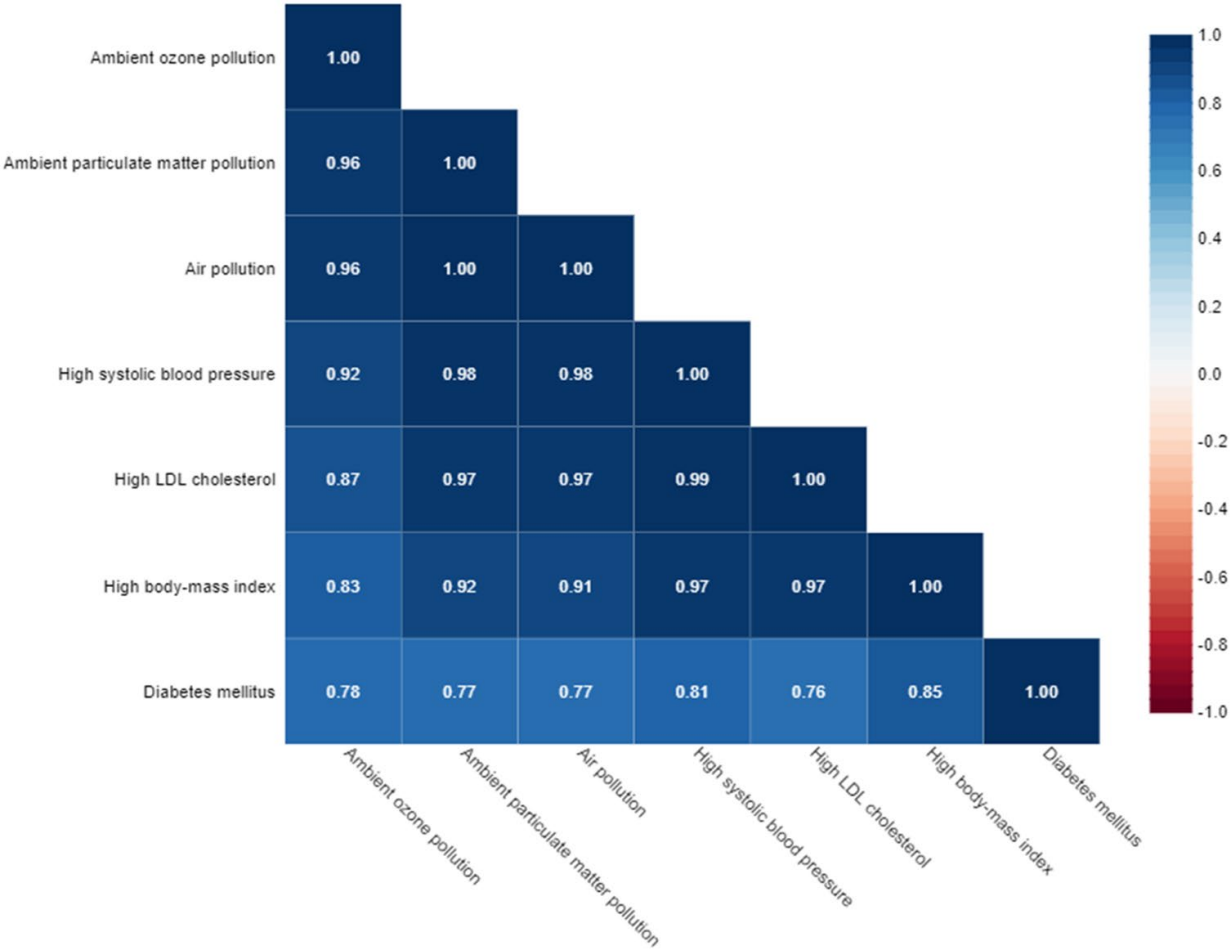
The relationship between the prevalence of DM2, high LDL cholesterol, high BMI, and high SBP (as CVD risk factors) with exposure to air pollution.

The relationship between the prevalence of CVD risk factors in the population over 50 years, including DM2, high SBP, high BMI, high LDL cholesterol, and AAP, was shown in Fig. 11. Analysis Spearman correlation displayed a significant relationship and positive correlation between air pollution and high SBP (p ≤ 0.001, r = 0.89), high LDL cholesterol (p ≤ 0.001, r = 0.87), high BMI (p ≤ 0.001, r = 0.83) and DM2 (p ≤ 0.001, r = 0.77).

There is a reciprocation interaction between air pollution and risk factors of CVD. Simultaneously, air pollutants may exacerbate and constantly stimulate several traditional risk factors^75,76^. All of which increase the incidence and severity of CVD. Hypertension is a complex disease with unknown causes. The positive relation between hypertension and CVD has been well demonstrated in some potential cohort research in developed and developing countries^77–79^. It influences almost one billion people worldwide and participates significantly in GBD and mortality^80^. Over recent years, human epidemiological investigations have assessed the relationship between long-term and short-term exposure to air pollution with hypertension and BP levels^81,82^. Exposure to AAP and potential environmental pollutants, including heavy metals, are associated with the prevalence of hypertension^83^. Numerous panel studies worldwide have also shown an association between concentrations of PM_2.5_, carbon black, and other pollutants with increased BP from a few hours to a couple of days^84,85^. Perhaps most important is prolonged exposure to PM_2.5_ and traffic-related emissions, which may increase chronic hypertension by themselves^86^. Smoking is additionally associated with high BP and CVD mortality^87,88^. While distinct evidence indicates that smoking and high BP affect the risk of death from CVD, the mixed impacts of these factors have rarely been examined. In Wold et al. study, It was indicated that systemic hypertension and vasoconstriction because of transient exposure to PM could increase the risk of acute heart failure. Besides, pulmonary and right ventricular diastolic pressures increment with PM exposure, showing the impact of pulmonary vasoconstriction on air pollution that can worsen congestive heart failure^89^.

Evidence suggests that despite the undeniable impact of poor eating habits with extra calorie intake and low physical activity^90^, endocrine-disrupting chemicals (EDC) also intervene in insulin secretion to demonstrate the importance of exposure to environmental pollutants on the pathogenesis of diabetes^91,92^. Air pollution may impair insulin response sensitivity^76^. In addition to hypertension and diabetes, obesity and overweight can participate in the global burden of chronic diseases such as CVD. Low-level exposures to industrial air pollution can play and be considered an endocrine disruptor in the obesity epidemic^93^. Obese people with diabetes might be at greater risk for cardiovascular impacts of PM_2.5_ exposure^7^.

Although not all studies confirm a positive correlation between exposure to AAP and the incidence of multifactorial diseases such as diabetes, hypertension, and obesity, public health outcomes indicate the potential risk of air pollution as a pervasive environmental risk factor for hypertension or diabetes^57^. However, the participatory role of these hemodynamic changes in expanding acute cardiovascular events associated with AAP is unclear^94,95^. Since the effect of these factors together on heart disease is not fully understood, laboratory studies seem to be useful to determine the mechanism of these effects.

### Strengths and limitations

Exposure to AAP is pervasive and occurs in a wide range. We were able to show this relationship to a large extent by considering other influential factors along with air pollution. In many cases, measuring the long-term exposure of each person to any of the air pollutant particles is very difficult and complex, and our use of an ecological study has been able to solve this problem to a large extent. This investigation inspected the relationship between AAP and CVD mortality, environmental exposure and risk factors for CVD (DM2, high BMI, high SBP, high LDL cholesterol), and the relationship between traditional risk factors and CVD prevalence mortality.

However, we know about the limitations of our study. This study is ecological, and the ecological fallacy should not be ignored^96,97^. The results of our study are the result of one data set and may not be generalizable to all individuals. Outdoor pollution may be approximately similar for residents of an area. However, indoor pollution can be quite different depending on the lifestyle of the people and the different ventilation of the house from one person to another. The GBD Study expresses that indoor air pollution represents 3 million deaths worldwide and is the fourth-highest risk factor for all deaths^50^. In addition, the cumulative effects of air pollutants should not be ignored. It should not be forgotten that air pollutants are varied during different days and seasons due to the temperature divergence. Personal and occupational exposure to air pollutants in vulnerable populations, particularly people with diabetes, high blood cholesterol, and high BMI could be reduced via simple measures, comprising:

- Preference for walking, cycling, and public transport over car or motorcycle,
- Avoid walking and cycling on high-traffic streets, especially during busy hours,
- Exercising in healthy environments such as parks and gardens,
- Considering the limitation of outdoor times during very polluted periods, particularly for infants, the elderly, and those with cardiorespiratory issues.

It is worth mentioning, that aside from preventive actions comprising indoor air purifications, smoking restrictions, newly generated and low emission cars, environmental researchers, have suggested large-scale outdoor devices for purifying urban air^98^. Burning fossil fuels is not just a significant source of air pollution yet additionally an important source of greenhouse gases. This way, avoiding the utilization of fossil fuels derivatives will have significant advantages for human health in terms of energy production, both through air pollution exposure and climate change.

## Conclusion

The present study investigated the relationship between exposure to ambient air pollution and CVD mortality and other risk factors for CVD mortality. Our study findings indicated a significant relationship between ambient air pollution, ambient PM pollution, ambient ozone, and CVD mortality. We showed the relationship between the exposure to AAP (PM, ozone, and total ambient air pollution, Separately) and each of the investigated traditional risk factors of CVD, including DM2, high SBP, high BMI, and high LDL cholesterol. There is strong evidence to support the mechanism of the relationship between AAP and CVD mortality. However, the level of indoor air pollution should not be ignored. Due to the widespread prevalence of air pollution exposure, political interventions to decrease environmental pollution, especially air pollution, can significantly affect CVD health for people. Air pollution should be one of the various significant modifiable risk factors in the prevention and organization of CVD in health and investigation priorities. Our study suggests that more research must be done on the ideal strategies for reducing AAP and the effects of these exposures on the incidence and mortality of CVD and other associated risk factors.

## Data Availability

All used raw data in this study are available at http://www.healthdata.org/. The datasets analysed during the current study are available from the corresponding author on reasonable request.

## References

[1] Franchini M, Mannucci PM (2012). Air pollution and cardiovascular disease. Thromb. Res..

[2] Langrish JP (2012). Reducing personal exposure to particulate air pollution improves cardiovascular health in patients with coronary heart disease. Environ. Health Perspect..

[3] Tanwar V, Katapadi A, Adelstein JM, Grimmer JA, Wold LE (2018). Cardiac pathophysiology in response to environmental stress: A current review. Curr. Opin. Physiol..

[4] Franchini, M. & Mannucci, P. M. Particulate air pollution and cardiovascular risk: short-term and long-term effects. in *Seminars in Thrombosis and Hemostasis*. Vol. 35. 665–670 (© Thieme Medical Publishers, 2009). 10.1055/s-0029-1242720 20013533

[5] Shah ASV (2013). Global association of air pollution and heart failure: A systematic review and meta-analysis. Lancet.

[6] Cesaroni G (2014). Long term exposure to ambient air pollution and incidence of acute coronary events: Prospective cohort study and meta-analysis in 11 European cohorts from the ESCAPE Project. BMJ.

[7] Brook RD (2010). Particulate matter air pollution and cardiovascular disease: An update to the scientific statement from the American Heart Association. Circulation.

[8] Héroux M-E (2015). Quantifying the health impacts of ambient air pollutants: Recommendations of a WHO/Europe project. Int. J. Public Health.

[9] An Z, Jin Y, Li J, Li W, Wu W (2018). Impact of particulate air pollution on cardiovascular health. Curr. Allergy Asthma Rep..

[10] Zanobetti A, Baccarelli A, Schwartz J (2011). Gene-air pollution interaction and cardiovascular disease: A review. Prog. Cardiovasc. Dis..

[11] Liang R (2014). Effect of exposure to PM2.5 on blood pressure: A systematic review and meta-analysis. J. Hypertens..

[12] Jerrett M (2014). Traffic-related air pollution and obesity formation in children: A longitudinal, multilevel analysis. Environ. Heal..

[13] McConnell R (2015). A longitudinal cohort study of body mass index and childhood exposure to secondhand tobacco smoke and air pollution: The Southern California Children’s Health Study. Environ. Health Perspect..

[14] Renzi M (2018). Air pollution and occurrence of type 2 diabetes in a large cohort study. Environ. Int..

[15] Jomova K (2011). Arsenic: Toxicity, oxidative stress and human disease. J. Appl. Toxicol..

[16] Al-Kindi SG, Brook RD, Biswal S, Rajagopalan S (2020). Environmental determinants of cardiovascular disease: Lessons learned from air pollution. Nat. Rev. Cardiol..

[17] Noroozian M (2012). The elderly population in iran: An ever growing concern in the health system. Iran. J. Psychiatry Behav. Sci..

[18] Chokshi DA, Farley TA (2012). The cost-effectiveness of environmental approaches to disease prevention. N. Engl. J. Med..

[19] Nieuwenhuijsen MJ (2018). Influence of urban and transport planning and the city environment on cardiovascular disease. Nat. Rev. Cardiol..

[20] Barnett AG (2006). The effects of air pollution on hospitalizations for cardiovascular disease in elderly people in Australian and New Zealand cities. Environ. Health Perspect..

[21] Koken PJM (2003). Temperature, air pollution, and hospitalization for cardiovascular diseases among elderly people in Denver. Environ. Health Perspect..

[22] Institute for Health Metrics and Evaluation. GBD 2019. (University of Washington, 2022).

[23] IHME. *GBD 2019 Data and Tools Overview*. (University of Washington, 2020).

[24] Dicker D (2018). Global, regional, and national age-sex-specific mortality and life expectancy, 1950–2017: A systematic analysis for the Global Burden of Disease Study 2017. Lancet.

[25] Miller DC, Salkind NJ (2002). Handbook of Research Design and Social Measurement.

[26] Rosenthal FS, Carney JP, Olinger ML (2008). Out-of-hospital cardiac arrest and airborne fine particulate matter: A case–crossover analysis of emergency medical services data in Indianapolis, Indiana. Environ. Health Perspect..

[27] Ensor KB, Raun LH, Persse D (2013). A case-crossover analysis of out-of-hospital cardiac arrest and air pollution. Circulation.

[28] Forastiere F (2005). A case-crossover analysis of out-of-hospital coronary deaths and air pollution in Rome, Italy. Am. J. Respir. Crit. Care Med..

[29] Levy D (2001). A case-crossover analysis of particulate matter air pollution and out-of-hospital primary cardiac arrest. Epidemiology.

[30] Silverman RA (2010). Association of ambient fine particles with out-of-hospital cardiac arrests in New York City. Am. J. Epidemiol..

[31] Naghavi M (2017). Global, regional, and national age-sex specific mortality for 264 causes of death, 1980–2016: A systematic analysis for the Global Burden of Disease Study 2016. Lancet.

[32] Berend N (2016). Contribution of air pollution to COPD and small airway dysfunction. Respirology.

[33] Vahedian, M., Khanjani, N., Mirzaee, M. & Koolivand, A. Associations of short-term exposure to air pollution with respiratory hospital admissions in Arak, Iran. *J. Environ. Health Sci. Eng.* **15**, 17 (2017). 10.1186/s40201-017-0277-z PMC5514473 28725443

[34] Yaser HS, Narges K, Yaser S, Rasoul M (2014). Air pollution and cardiovascular mortality in Kerman from 2006 to 2011. Am. J. Cardiovasc. Dis. Res..

[35] Khaefi M (2017). Association of particulate matter impact on prevalence of chronic obstructive pulmonary disease in Ahvaz, southwest Iran during 2009–2013. Aerosol Air Qual. Res..

[36] Khaniabadi YO (2017). Exposure to PM10, NO_2_, and O_3_ and impacts on human health. Environ. Sci. Pollut. Res. Int..

[37] Momtazan, M. *et al.* An investigation of particulate matter and relevant cardiovascular risks in Abadan and Khorramshahr in 2014–2016. *Toxin Rev.* **38**, 1–8 (2018).

[38] Martinelli N, Olivieri O, Girelli D (2013). Air particulate matter and cardiovascular disease: A narrative review. Eur. J. Intern. Med..

[39] Khaniabadi YO (2019). Mortality and morbidity due to ambient air pollution in Iran. Clin. Epidemiol. Glob. Health.

[40] Almeida-Silva M (2015). Exposure and dose assessment to particle components among an elderly population. Atmos. Environ..

[41] Suh HH, Zanobetti A, Schwartz J, Coull BA (2011). Chemical properties of air pollutants and cause-specific hospital admissions among the elderly in Atlanta, Georgia. Environ. Health Perspect..

[42] Chien L-C, Yang C-H, Yu H-L (2012). Estimated effects of Asian dust storms on spatiotemporal distributions of clinic visits for respiratory diseases in Taipei children (Taiwan). Environ. Health Perspect..

[43] Khaniabadi YO (2018). Chronic obstructive pulmonary diseases related to outdoor PM10, O_3_, SO_2_, and NO_2_ in a heavily polluted megacity of Iran. Environ. Sci. Pollut. Res..

[44] Omidi Khaniabadi, Y. *et al.* Air quality modeling for health risk assessment of ambient PM10, PM2.5 and SO_2_ in Iran. *Hum. Ecol. Risk Assess. Int. J.* **25**, 1298–1310 (2019).

[45] Newell K, Kartsonaki C, Lam KBH, Kurmi OP (2017). Cardiorespiratory health effects of particulate ambient air pollution exposure in low-income and middle-income countries: A systematic review and meta-analysis. Lancet Planet. Health.

[46] Dominici F (2006). Fine particulate air pollution and hospital admission for cardiovascular and respiratory diseases. JAMA.

[47] Qiu H (2022). Inflammatory and oxidative stress responses of healthy elders to solar-assisted large-scale cleaning system (SALSCS) and changes in ambient air pollution: A quasi-interventional study in Xi’an, China. Sci. Total Environ..

[48] Fiordelisi A (2017). The mechanisms of air pollution and particulate matter in cardiovascular diseases. Heart Fail. Rev..

[49] Yang, D., Yang, X., Deng, F. & Guo, X. Ambient air pollution and biomarkers of health effect. *Ambient Air Pollut. Health Impact China* **1017**, 59–102 (2017). 10.1007/978-981-10-5657-4_4 29177959

[50] Lim SS (2012). A comparative risk assessment of burden of disease and injury attributable to 67 risk factors and risk factor clusters in 21 regions, 1990–2010: A systematic analysis for the Global Burden of Disease Study 2010. Lancet.

[51] Newby DE (2015). Expert position paper on air pollution and cardiovascular disease. Eur. Heart J..

[52] Brook RD, Newby DE, Rajagopalan S (2018). Air pollution and cardiometabolic disease: An update and call for clinical trials. Am. J. Hypertens..

[53] Kang S-H (2016). Ambient air pollution and out-of-hospital cardiac arrest. Int. J. Cardiol..

[54] Thurston GD (2016). Ambient particulate matter air pollution exposure and mortality in the NIH-AARP diet and health cohort. Environ. Health Perspect..

[55] Gallagher LG (2010). Applying a moving total mortality count to the cities in the NMMAPS database to estimate the mortality effects of particulate matter air pollution. Circulation.

[56] Rodopoulou S, Samoli E, Chalbot M-CG, Kavouras IG (2015). Air pollution and cardiovascular and respiratory emergency visits in Central Arkansas: A time-series analysis. Sci. Total Environ..

[57] Teng T-HK (2014). A systematic review of air pollution and incidence of out-of-hospital cardiac arrest. J. Epidemiol. Commun. Health.

[58] Brook RD (2004). Air pollution and cardiovascular disease: A statement for healthcare professionals from the expert panel on population and prevention science of the American Heart Association. Circulation.

[59] Raza A (2014). Short-term effects of air pollution on out-of-hospital cardiac arrest in Stockholm. Eur. Heart J..

[60] Baccarelli A (2007). Effects of exposure to air pollution on blood coagulation. J. Thromb. Haemost..

[61] Franchini M, Mannucci PM (2011). Thrombogenicity and cardiovascular effects of ambient air pollution. Blood.

[62] Yin F (2013). Diesel exhaust induces systemic lipid peroxidation and development of dysfunctional pro-oxidant and pro-inflammatory high-density lipoprotein. Arterioscler. Thromb. Vasc. Biol..

[63] Chirinos JA (2005). Elevation of endothelial microparticles, platelets, and leukocyte activation in patients with venous thromboembolism. J. Am. Coll. Cardiol..

[64] Adar SD (2013). Fine particulate air pollution and the progression of carotid intima-medial thickness: A prospective cohort study from the multi-ethnic study of atherosclerosis and air pollution. PLoS Med..

[65] Kampfrath T (2011). Chronic fine particulate matter exposure induces systemic vascular dysfunction via NADPH oxidase and TLR4 pathways. Circ. Res..

[66] Sun Q (2005). Long-term air pollution exposure and acceleration of atherosclerosis and vascular inflammation in an animal model. JAMA.

[67] Dennekamp, M. *et al.* Outdoor air pollution as a trigger for out-of-hospital cardiac arrests. *Epidemiology* **21**, 494–500 (2010). 10.1097/EDE.0b013e3181e093db 20489649

[68] Straney L (2014). Evaluating the impact of air pollution on the incidence of out-of-hospital cardiac arrest in the Perth Metropolitan Region: 2000–2010. J. Epidemiol. Commun. Health.

[69] Sullivan J (2003). Exposure to ambient fine particulate matter and primary cardiac arrest among persons with and without clinically recognized heart disease. Am. J. Epidemiol..

[70] Barton TJ (2021). Traditional cardiovascular risk factors strongly underestimate the 5-year occurrence of cardiovascular morbidity and mortality in spinal cord injured individuals. Arch. Phys. Med. Rehabil..

[71] Burg MM (2017). Risk for incident hypertension associated with PTSD in military veterans, and the effect of PTSD treatment. Psychosom. Med..

[72] Hinojosa R (2019). Veterans’ likelihood of reporting cardiovascular disease. J. Am. Board Fam. Med..

[73] Rush T, LeardMann CA, Crum-Cianflone NF (2016). Obesity and associated adverse health outcomes among US military members and veterans: Findings from the millennium cohort study. Obesity.

[74] Stefanovics EA, Potenza MN, Pietrzak RH (2020). Smoking, obesity, and their co-occurrence in the US military veterans: Results from the national health and resilience in veterans study. J. Affect. Disord..

[75] Brook RD (2009). Insights into the mechanisms and mediators of the effects of air pollution exposure on blood pressure and vascular function in healthy humans. Hypertension.

[76] Rajagopalan S, Brook RD (2012). Air pollution and type 2 diabetes: Mechanistic insights. Diabetes.

[77] Franklin SS, Wong ND (2013). Hypertension and cardiovascular disease: Contributions of the Framingham Heart Study. Glob. Heart.

[78] Gu D (2008). Blood pressure and risk of cardiovascular disease in Chinese men and women. Am. J. Hypertens..

[79] Wang H (2010). Blood pressure, body mass index and risk of cardiovascular disease in Chinese men and women. BMC Public Health.

[80] O’Brien E (2017). The Lancet Commission on hypertension: Addressing the global burden of raised blood pressure on current and future generations. J. Clin. Hypertens..

[81] Cai Y (2016). Associations of short-term and long-term exposure to ambient air pollutants with hypertension: A systematic review and meta-analysis. Hypertension.

[82] Zhang Z, Laden F, Forman JP, Hart JE (2016). Long-term exposure to particulate matter and self-reported hypertension: A prospective analysis in the Nurses’ Health Study. Environ. Health Perspect..

[83] Cosselman KE, Navas-Acien A, Kaufman JD (2015). Environmental factors in cardiovascular disease. Nat. Rev. Cardiol..

[84] Baccarelli A (2011). Effects of particulate air pollution on blood pressure in a highly exposed population in Beijing, China: A repeated-measure study. Environ. Heal..

[85] Mordukhovich I (2009). Black carbon exposure, oxidative stress genes, and blood pressure in a repeated-measures study. Environ. Health Perspect..

[86] Chen H (2014). Spatial association between ambient fine particulate matter and incident hypertension. Circulation.

[87] Honjo K (2010). The effects of smoking and smoking cessation on mortality from cardiovascular disease among Japanese: Pooled analysis of three large-scale cohort studies in Japan. Tob. Control.

[88] Lawlor DA, Song Y-M, Sung J, Ebrahim S, Smith GD (2008). The association of smoking and cardiovascular disease in a population with low cholesterol levels: A study of 648 346 men from the Korean national health system prospective cohort study. Stroke.

[89] Wold LE (2012). Cardiovascular remodeling in response to long-term exposure to fine particulate matter air pollution. Circ. Hear. Fail..

[90] Zoeller RT (2012). Endocrine-disrupting chemicals and public health protection: A statement of principles from The Endocrine Society. Endocrinology.

[91] Ruiz D, Becerra M, Jagai JS, Ard K, Sargis RM (2018). Disparities in environmental exposures to endocrine-disrupting chemicals and diabetes risk in vulnerable populations. Diabetes Care.

[92] Taylor D (2014). Toxic Communities: Environmental Racism, Industrial Pollution, and Residential Mobility.

[93] Newbold RR, Padilla-Banks E, Jefferson WN (2009). Environmental estrogens and obesity. Mol. Cell. Endocrinol..

[94] Szyszkowicz M, Rowe BH, Brook RD (2012). Even low levels of ambient air pollutants are associated with increased emergency department visits for hypertension. Can. J. Cardiol..

[95] van den Hooven EH (2011). Air pollution, blood pressure, and the risk of hypertensive complications during pregnancy: The generation R study. Hypertension.

[96] Vali, M. *et al.* Effect of meteorological factors and Air Quality Index on the COVID-19 epidemiological characteristics: An ecological study among 210 countries. *Environ. Sci. Pollut. Res.* **38**, 1–11 (2021). 10.1007/s11356-021-14322-6 PMC8140752 34024000

[97] Kiani B (2021). Association between heavy metals and colon cancer: An ecological study based on geographical information systems in North-Eastern Iran. BMC Cancer.

[98] Cyranoski D (2018). China tests giant air cleaner to combat smog. Nature.

